# Synthesis and Characterization of Agarose Hydrogels for Release of Diclofenac Sodium

**DOI:** 10.3390/ma16176042

**Published:** 2023-09-02

**Authors:** Anna Jarosz, Oliwia Kapusta, Dorota Gugała-Fekner, Mariusz Barczak

**Affiliations:** Faculty of Chemistry, Institute of Chemical Sciences, Maria Curie-Sklodowska University, Maria Curie-Sklodowska Sq. 3, 20-031 Lublin, Poland; aniaa.jarosz@wp.pl (A.J.); dorota.gugala-fekner@mail.umcs.pl (D.G.-F.)

**Keywords:** agarose, hydrogel, controlled release, drug release, diclofenac sodium

## Abstract

Hydrogels are attractive biomaterials for the controlled release of various pharmaceuticals, due to their ability to embed biologically active moieties in a 3D polymer network. Among them, agarose-based hydrogels are an interesting, but still not fully explored, group of potential platforms for controlled drug release. In this work, agarose hydrogels with various contents of citric acid were prepared, and their mechanical and physicochemical properties were investigated using various instrumental techniques, such as rheological measurements, attenuated total reflection–Fourier transform infrared spectroscopy (ATR-FTIR). Releasing tests for diclofenac sodium (DICL) were run in various environments; water, PBS, and 0.01 M NaOH; which remarkably affected the profile of the controlled release of this model drug. In addition to affecting the mechanical properties, the amount of citric acid incorporated within a hydrogel network during synthesis was also of great importance to the rate of DICL release. Therefore, due to their high biocompatibility, agarose hydrogels can be regarded as safe and potential platforms for controlled drug release in biomedical applications.

## 1. Introduction

Biomedical applications of hydrogels have been constantly evolving in recent years, driven by new discoveries in biology and chemistry. The use of new methods in the process of obtaining these materials has resulted in the obtention of highly functional and complex composite hydrogels containing various nano and microstructures, which can be used in the pharmaceutical industry, and in many areas of biomedicine, e.g., in controlled drug delivery systems [1,2,3,4], tissue engineering [5,6,7], 3D printing [8,9,10], or biosensors [11,12,13]. 

The conventional administration of drugs for the treatment of various diseases may often lead to adverse and toxic side effects, due to a too high-plasma concentration, and distribution of the drug substance to healthy tissues. To overcome this problem, appropriate delivery systems should be used to enable a controlled and continuous drug release to the target site. In recent years, significant efforts have been made to exploit the potential of hydrogels in drug delivery, as hydrogels can offer a suitable way to supply low- or high-molecular-weight pharmaceuticals and other bioactive agents at a specific location and controlled time [2,14,15,16]. These systems offer many benefits, mainly focusing on improving the safety and effectiveness of drugs, but also ensuring targeted drug delivery, improving bioavailability, extending drug action, and improving the stability of therapeutic agents against chemical or enzymatic degradation [17]. 

Polysaccharide polymers are one of the most important materials used in the design and fabrication of efficient drug carriers, and have the potential to meet a wide range of needs. Common polycarbohydrate polymers, such as chitosan, starch, and cellulose, are often used in drug delivery systems but, unfortunately, they also have many disadvantages. For example, natural chitosan is soluble only in a diluted, acidic, aqueous solution, and loses its mechanical properties in an alkaline environment with a higher pH, while starch has generally weak mechanical properties, and cellulose is insoluble in both polar and non-polar solvents [18,19,20]. In contrast, agarose, with its low toxicity, ability to become a hydrogel, appropriate viscoelasticity, and thermal reversibility is a common substrate in the construction of capsules, and is a very good carrier of drugs in their controlled release. In addition, the high biocompatibility of agarose has been repeatedly confirmed in many biomedical applications, including controlled drug delivery and tissue engineering [21,22,23].

Agarose is a natural, plant-derived polysaccharide obtained from red marine algae (*Rhodophyceae*), and is composed of (1–3)-linked agarobiose units of β-D-galactopyranose (1–4)-linked to 3,6-anhydro-α-L-galactopyranose, which are components of galactan [24,25]. The process of hydrogel formation very quickly undergoes a sol–gel transition upon cooling, and forms a three-dimensional network, where single agarose chains first form double helices stabilized by hydrogen bonds. Further cooling leads to the aggregation of the formed double helices, resulting in physically cross-linked agarose hydrogels. The important advantage of this process is that the physically formed hydrogels do not require the use of additional cross-linking agents; however, inducing chemical cross-linking may result in better mechanical properties. Due to the dense three-dimensional network and the fast kinetics of the gelling process, agarose may form durable, but highly elastic, gels [26]. 

The unique properties of agarose, such as its non-toxicity, low cost, and ease of controllable gelation, mean that it is commonly used in biomedicine fields, such as cell therapy, the sustained release of medicinal substances, and tissue engineering. Thanks to a high biocompatibility, agarose-based hydrogels can be used inside the body without a significant response from the immune system.

Agarose hydrogels are widely used in drug delivery systems due to their biocompatibility and solute permeability. Drug release systems can be built using the biocompatibility of agarose, through enclosing active ingredients in agarose hydrogel nanoparticles. For example, Grolman et al. obtained a 0.5% agarose hydrogel with a high content of minocycline and gentamicin, i.e., strong bactericidal antibiotics [27]. The tests showed that both antibiotics remained stable in the hydrogel matrix, and could be released in a stable and controlled manner. As a consequence, an effective system was obtained that reduced the amount of bacteria in burn wounds with a similar effect to a commonly used ointment with silver sulfadiazine. Agarose loaded with gentamicin was also studied by Soylu et al. who, additionally, enriched them with tannic acid and calcium chloride as cross-linking agents [28]. The agarose hydrogels obtained in this way had promising antibacterial properties, and could be used in the surface modification process of spinal implants, which are used to treat chronic diseases such as vertebral fractures, spondylitis, and scoliosis. 

Agarose can also be mixed with other polysaccharides, peptides, and magnetic nanoparticles, to form more complex platforms with improved physicochemical properties. In addition, agarose gels can be customized in terms of their pore size, structure, and function, via proper adjustment of the concentration of agarose and tailored chemical modifications, to create a versatile drug delivery system. Yiluo et al. obtained an agarose/succinoglycan hydrogel that could respond to pH changes, and tested it for use as a drug carrier [29]. The tests showed that the modified hydrogel had significantly improved properties, such as a high flexibility, thermostability, and porosity, compared to the classic agarose hydrogel. In addition, this hydrogel showed the ability to control the release of ciprofloxacin depending on the pH value: with an increasing pH, it released an increasing amount of the drug. Aslam et al. reported a pH-sensitive cross-linked agarose hydrogel with added Pluronic block co-polymer, glutaraldehyde, and methacrylic acid, via free radical polymerization [30]. It was shown that the obtained hydrogel was able to release cyclophosphamine in a controlled manner for 24 h, while no toxicity effects to the eyes, skin, or mouth were observed. 

In this work, agarose hydrogels with various contents of citric acid were synthesized and characterized, for the evaluation of the effect of the citric acid contents on the mechanical and physicochemical properties, as well as the controlled release of diclofenac sodium (DICL, used here as a model drug), in various environments. Two green, biocompatible, biodegradable, cheap and abundant compounds (agarose and citric acid) have been employed in the preparation of the obtained platforms, to induce suitable properties [31]. 

## 2. Materials and Methods

### 2.1. Reagents

The following reagents were used as received: agarose, AG (Merck, Rahway, NJ, USA), citric acid, CA (POCH, Gliwice, Poland), NaOH (POCH, Poland), diclofenac sodium salt, DICL (>98%, Sigma-Aldrich, St. Louis, MO, USA), phosphate-buffered saline tablets (PBS, Life Technologies Ltd., Renfrew, UK). All chemicals were used as received, without further purification.

### 2.2. Preparation of the Agarose Hydrogels

To prepare 25 mL of a 2% agarose solution, a 0.5 g portion of agarose powder was added to 24.5 mL of distilled water, closed in a sealed vessel, and placed for 1 h in a water bath at 70 °C, while stirring (350 rpm). Then, 4 mL of the obtained agarose solution was poured into prepared glass vials. Immediately thereafter, 1 mL of water or citric acid was added, and vigorously vortexed for 30 s. From the solutions prepared in this way, several 1.5 mL volumes were pipetted, and placed in plastic cassettes. To obtain reliable and reproducible results, three identical hydrogels were always prepared, each of them subjected to the same synthesis protocol. The volume and concentration of the added solutions are shown schematically in Table 1, along with the names of the resulting hydrogels.

To prepare the hydrogels loaded with diclofenac sodium (DICL), the same protocol was used. Before the hydrogels were pipetted out into the plastic cassettes, they were premixed with 0.2% diclofenac solution (1:1 volume ratio). The resulting DICL-loaded hydrogels were named by adding the letter L to the end of each name. Therefore, for example, DICL-loaded hydrogel AG-1L was obtained in the same way as unloaded hydrogel AG-1, but it was simultaneously loaded with DICL. The contents of DICL in the obtained loaded hydrogels were as follows: AG-0: 1.87%, for AG-1: 1.67%, for AG-2: 1.43%, for AG-3: 1.15%, AG-4: 0.83%.

### 2.3. Characterization of the Physicochemical Properties of the Hydrogels

Water-releasing tests were carried out for each hydrogel. Freshly obtained hydrogels with a predetermined weight (in the range between 1.54 and 1.65 g) were placed onto a glass slide, and submitted to drying at 40 °C (for the first 270 min), and then the process was accelerated via raising the temperature to 60 °C. Weight losses were recorded during drying at specific times during 15 h (i.e., until only dry residue remained, and the weight did not change over time anymore).

Scanning electron microscopy (SEM) images of the dehydrated hydrogels were collected on the Quanta 3DFEG (Thermo Fisher Scientific/FEI, Waltham, MA, USA) microscope, with the accelerating voltage 5 keV.

The ATR-FTIR spectra were measured by means of the FTIR 6200 spectrophotometer (Jasco, Oklahoma, OK, USA) in the range of 4000–400 cm^−1^, with the resolution 4 cm^−1^, via averaging 32 scans. After that, the spectra were analyzed using Spectra Manager v2.0 software.

### 2.4. Rheological Measurements

The rheological properties of the obtained agarose hydrogels were run at RT, using the MCR 302e magneto rheometer (Anton Paar, Graz, Austria), with a plate–plate geometry (Φ = 20 mm). The linear viscoelastic region (LVR) of the studied hydrogels was determined during amplitude sweep tests, at a constant frequency of 1 Hz, and stepwise increasing shear strain amplitude, γ_0_. The values of the storage and loss moduli (G′ and G″, respectively) were derived as functions of γ_0_. The frequency sweep tests were run at a fixed shear strain amplitude (γ_0_ = 0.03% i.e., within the LVR region), and frequency increasing from 1 to 16 Hz. The values of G′ and G″ were derived as functions of the frequency [32].

### 2.5. Release Tests of Diclofenac Sodium

The release of DICL was accomplished through the immersion of the DICL-loaded hydrogels in 25 mL of selected media, i.e., 0.01 M HCl, water, unbuffered PBS solution, and 0.01 M NaOH. The release of diclofenac was monitored spectrophotometrically, using the UV–Vis spectrometer Specord 200 (Analytic Jena, Jena, Germany), via measuring the absorbance at λ_max_ = 278 nm, which is characteristic of DICL [33,34,35]. The release experiments were carried out until a plateau was observed in the release curves, which was a sign that no more DICL would be able to be released from the tested hydrogels.

## 3. Results

### 3.1. Dehydration Studies

**Figure 1:**
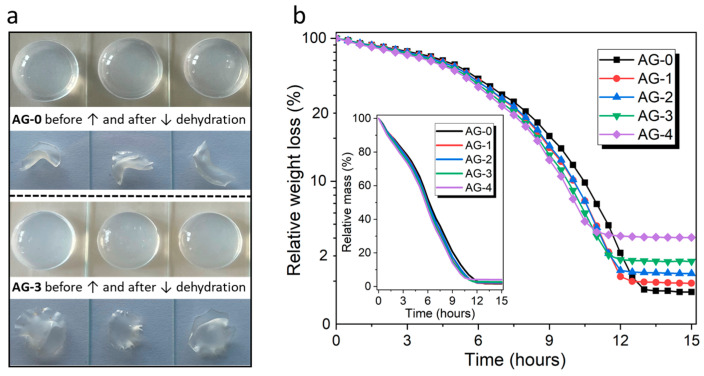
(**a**) Example photos of hydrated and dehydrated hydrogels, and (**b**) the dehydration profiles of the studied hydrogels (the inset box shows the weight losses on a linear scale).

All the obtained hydrogels formed a very homogeneous structure, and it was impossible to distinguish individual samples that differed in the content of citric acid using the naked eye. Example photos of hydrogels are shown in Figure 1a. Our research hypothesis assumed that the addition of citric acid should affect the properties of the resulting agarose hydrogels. In the first place, we wanted to check whether the addition of citric acid could affect the dehydration process of fresh hydrated hydrogels submitted to drying; the obtained results are presented in Figure 1b. 

As can be seen, the loss of water over time is almost linear, and similar for all the hydrogels, which lose water relatively evenly and at a similar rate. However, a closer analysis of the results (aligned using a logarithmic scale for weight losses) reveals some differences in the course of the dehydration process, which are revealed over time. The moment when the hydrogel is completely dehydrated depends on the amount of citric acid used during synthesis. For the AG-4 hydrogel, dehydration occurs the fastest among all hydrogels, i.e., after 11 h. For the AG-3 hydrogel, the time needed for full dehydration is 11.5 h; for AG-2, it is 12 h; and for AG-1, it is 12.5 h; while for AG-0, it is 13 h. Therefore, it can be assumed that the addition of citric acid causes faster dehydration, which may be related to the formation of a more cross-linked and rigid structure, with poorly adaptable porosity during water release. Therefore, water is released more easily from this rigid network, in contrast to more flexible, less cross-linked structures with more adaptable porosity for hydrogels that does not contain citric acid (AG-0) or contain its smaller amounts (AG-1, AG-2).

### 3.2. FTIR

**Figure 2:**
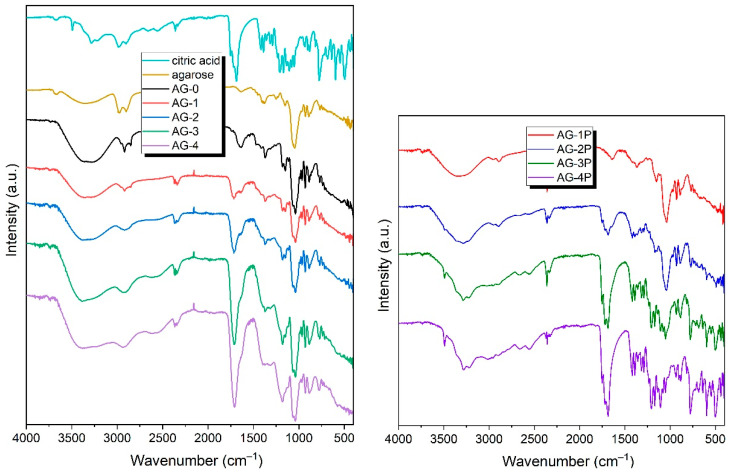
The FTIR spectra of the obtained hydrogels, along with the spectra of the initial compounds used to synthesize hydrogels: agarose and citric acid (**left** panel), and the spectra of mixed agarose and citric acid in proportions corresponding to those in the final hydrogels AG-1–AH-4 (**right** panel).

To investigate the chemical composition of the obtained hydrogels, ATR-FTIR spectra were obtained, and are presented in Figure 2. As can be seen, the FTIR spectra of the obtained hydrogels are quite similar, and contain a number of signals common to all the hydrogels AG-0–AG-4. In the range of ~3700–3000 cm^−1^, there is a wide absorption band, originating from the vibrations of the O-H groups present in the agarose structure, but also coming from the physically adsorbed water (which is unavoidable, even for dry samples) [36]. Interestingly, this already-wide band becomes even wider with the increasing content of citric acid. This can be clearly seen when comparing the hydrogels AG-0 and AG-4: for the former, the band extends to ~3000 cm^−1^; and for the latter, up to ~2200 cm^−1^. Such a significant broadening arises from the presence of the dimerized form of the carboxylic acid forming very strong hydrogen bonds, albeit with dynamically variable strengths; as a consequence of this phenomenon, the band is extended to the observed values. The gradual extension of this broad signal confirms that the hydrogels contain increasing amounts of citric acid.

In the range of ~3000–2800 cm^−1^, bands originating from the stretching vibrations of the –CH and –CH_2_ groups present in the agarose structure and citric acid are observed. In the case of the pure agarose-based hydrogel AG-0, the signals are clearly separated from each other, while, as the citric acid concentration increases, they merge into one wide signal, with its maximum at ~2950 cm^−1^. The band at ~1640 cm^−1^ can be attributed to the deformation vibrations of water. This band gradually starts to partially overlap with the band resulting from the stretching vibration of the C=O group, with its maximum at ~1715 cm^−1^.

The spectra also show a number of signals characteristic of agarose; these are: a band located at ~1365 cm^−1^ caused by C–C bending vibrations; a band located at ~1033 cm^−1^ caused by C–O stretching vibrations; bands at 1078 cm^−1^ and 930 cm^−1^, which are characteristic of a glycosidic bond; and a band located at ~890 cm^−1^, which can be attributed to the vibration of the C–O–C bridge of 3,6-anhydrogalactose [37,38].

On the spectra of all hydrogels, there is a lack of the two characteristic bands of citric acid, located at 1690 cm^−1^ and 1753 cm^−1^, and assigned to the C=O stretching of citric acid [39]. This may suggest that the cross-linking of citric acid with the agarose occurred. An almost inappreciable shoulder, located at ~1740 cm^−1^, can be related to the residual citric acid. Similar observations were discussed previously by Uranga et al. [40]. Comparing the spectra of the AG-1–AG-4 hydrogels with the corresponding mixtures AG-1P–AG-4P, which contain exactly the same proportion of agarose and citric acid, one can see that the spectra of the resulting hydrogels do not exhibit bands in the range of 400–800 cm^−1^ present in the citric acid itself and, of course, in the powdered mixtures. This may also indirectly indicate that the citric acid in the final hydrogels does not have the same form as in the mixture before the reaction.

### 3.3. SEM Imaging

**Figure 3:**
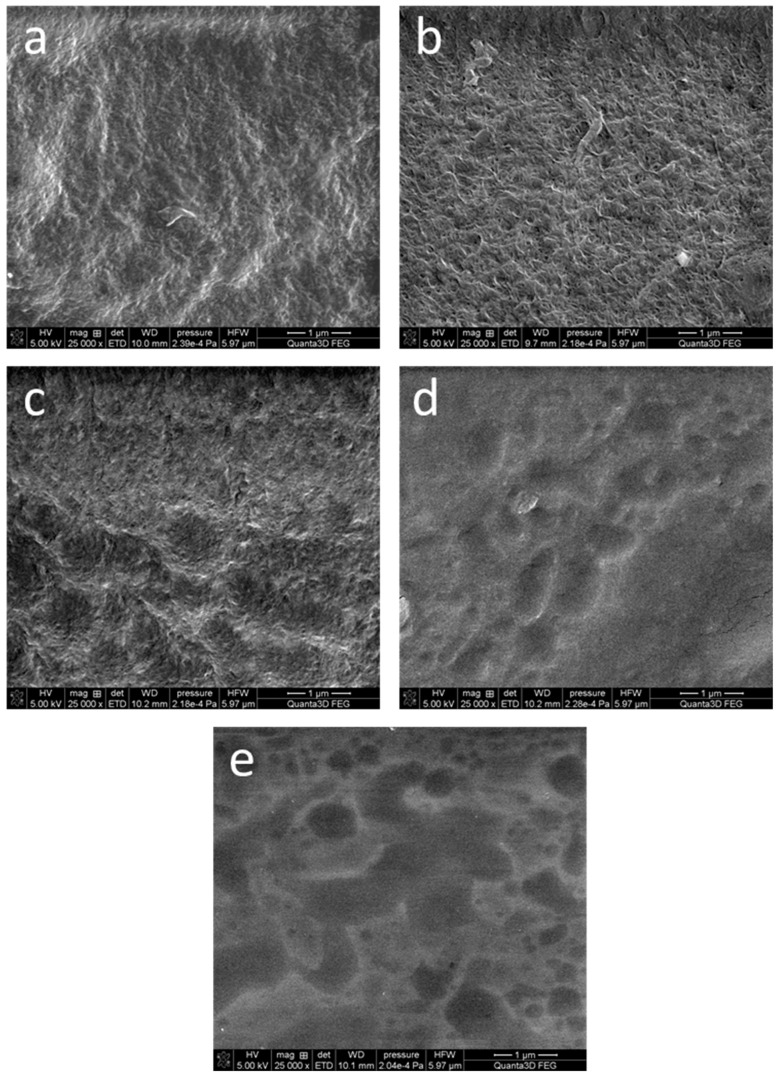
SEM microphotographs of the dehydrated hydrogels: (**a**) AG-0, (**b**) AG-1, (**c**) AG-2, (**d**) AG-3, (**e**) AG-4. The scale bar visible in the bottom right corner of each image is 1 μm.

Figure 3 shows representative photographs of the dehydrated hydrogels. The SEM images of the samples AG-0, AG-1, and AG-2 are quite similar, and show the fibrillar structure of the obtained hydrogels. However, in the case of the samples AG-3 and AG-4, this structure seems to be less visible, probably due to the higher content of citric acid. However, these photographs should be interpreted with caution, because these hydrogels were easily destroyed under electron beam irradiation. The received images are quite similar to the images obtained using cryo-SEM for hydrogels obtained from agarose with the same concentration as in our work [41].

### 3.4. Rheological Measurements

**Figure 4:**
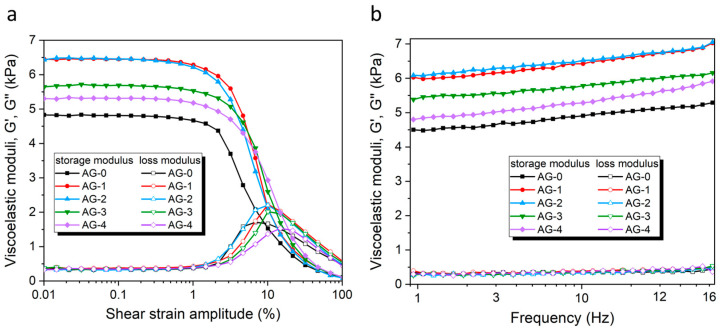
(**a**) The storage and loss moduli of the studied hydrogels as a function of the shear strain amplitude. (**b**) The storage and loss moduli of the studied hydrogels as a function of the frequency.

The rheological characteristics of the studied agarose hydrogels AG-0–AG-4 were tested using well-known protocols [32]. The dependence of the storage modulus (G′) and loss modulus (G″) as a function of the shear strain amplitude (SSA) in the oscillatory regime (ν = 1 Hz) is shown in Figure 4a. This dependence has a shape typical for a viscoelastic solid-like material, and is by characterized by G′ >> G″ at a low strain amplitude, which demonstrates the solid-like response of hydrogels [42]. Both viscoelastic moduli have a broad plateau-like region within the range from ~0.01–0.5% of the SSA, called the linear viscoelastic region (LVR). Thus, all the obtained hydrogels exhibit, unsurprisingly, an elastic dominated behavior (gel-like or solid structure) in the LVE range. The limit of the LVE range (called the yielding point) is also similar for all the hydrogels, and is ~0.5%.

When the SSAs reach 1%, the values of G′ decrease dramatically, whereas the G″ values first increase, reaching a maximum around 10% of the SSA, and then decrease again. The increase in G″ represents an enhancement in the dissipation of energy related to the irreversible destruction of the microstructure of the hydrogel by the shear forces. The appearing G″ maximum approximately coincides with the intersection of both curves (i.e., G′ and G″), with G″ being higher than the G′ above this maximum point, which represents a liquid-like behavior. The region where the G′ and G″ experience rapid changes is called a nonlinear viscoelastic region (NVR). Within this region, the irreversible deformation of the hydrogel internal structure occurs, and this decrease in elasticity is manifested via a tremendous decrease in the storage modulus, G′. At the microscopic level, the increase in the loss modulus, G″, reflects an increasing friction between the hydrogel chains/segments, as well as possible breakage in the agarose segments [43]. 

The observed differences in the G′ values between synthesized hydrogels are significant (cf. Table 2). As the rheological analyses of different samples of the same hydrogel gave convergent results, the observed differences between the hydrogels do not result from intra-group variance, but from an external factor causing these changes. The only variable during synthesis was the addition of various amounts of citric acid, so this may be the only factor influencing the mechanical properties. The hydrogel obtained without the addition of citric acid (AG-0) has G′ = 4815 Pa. The addition of relatively small amounts of citric acid means that the corresponding AG-1 and AG-2 hydrogels have significantly higher G′ values (6450 Pa and 6463 Pa, respectively). This strongly suggests that citric acid causes additional agarose cross-linking, resulting in increased G′ values. Interestingly, the addition of larger amounts of citric acid (which is the case with the AG-3 and AG-4 hydrogels) does not cause further increases in the G′ but, rather, its decrease in the AG-3 and AG-4 hydrogels (5682 Pa and 5313 Pa, respectively) when compared with the values for AG-1 and AG-2. In this case, the high content of citric acid no longer has an important effect on the agarose cross-linking (vide Figure 2), because its major part is only physically dispersed in the hydrogel, without chemical interactions with the polymer network. Therefore, a too-high content of citric acid not only does not improve mechanical properties, but it can even lower them, compared with the hydrogel where the amount of citric acid is optimal from the point of view of the maximum esterification efficiency (ideally, all the citric acid molecules have formed ester bonds with the agarose polymer chains, and there are no more opportunities to form ester bonds).

The dependence of the storage (G′) and loss (G″) moduli on the frequency at constant strain (γ = 0.03%, i.e., within the LVR) is presented in Figure 4b. Both moduli, the G′ and G″, increase with the frequency of oscillation for the range of frequencies under study (1–16 Hz). In the case of the G′, its increase is quite high, and continuous, in the tested frequency range, while in the case of the G″, there is only a slight, but noticeable, increase. This can be explained by increased chain interactions as the frequency increases, leading to collisions with the neighboring chains and, thus, causing a change in both moduli [25]. In all cases, the values of G′ were considerably larger than the corresponding values of G″. The G″ values were the same for all the hydrogels in the entire frequency range tested, while the G″ values show similar relative relationships, as in the case of their dependence on the SSC in LVR (cf. Figure 4a). The observed tendencies are typical of cross-linked polymer systems [44], as well as of soft human tissues [45].

The position of the yielding point, γ_L_ (i.e., the point of intersection of the G′ and G′ curves) is shifting toward higher oscillatory shear strain values (cf. Table 2). These results most probably indicate that interactions between the agarose and citric acid may reduce the aggregation of the helical structures of the agarose, leading to the formation of more flexible hydrogels. A similar effect was observed previously by Hu et al. for agarose/succinoglycan hydrogels [29].

### 3.5. Release of Diclofenac Sodium (DICL)

The releasing test for DICL (chosen here as a model drug) was performed in various solutions, ranging from 0.01 M HCl (pH = 2.0) via water (pH = 6.0) and PBS (pH = 7.2), to 0.01 M NaOH (pH = 12.0). Such a wide selection of media with different pH values was dictated, on the one hand, by scientific curiosity and, on the other hand, to cover the widest pH range. However, the most important criterion was that, among the selected model media, there should be cases that simulate various body fluids. For example, 0.01 M HCl simulates gastric juice well (pH~2), water simulates saliva (pH~6.5), and PBS simulates several important biological fluids, including blood and synovial fluid [46].

The most important observation was that all the hydrogels retained their integrity after the releasing tests, showing an excellent mechanical resistance, regardless of the medium in which were immersed (sometimes for many days).

The release of diclofenac sodium at an acidic pH (0.01 M HCl, pH = 2) was, expectably, negligible. This is due to the fact that diclofenac sodium has a pKa~4.2 [47,48] and, below this pH, it exists predominantly as undissociated acid, hardly soluble in water; therefore, there is no DICL release observed in water systems.

**Figure 5:**
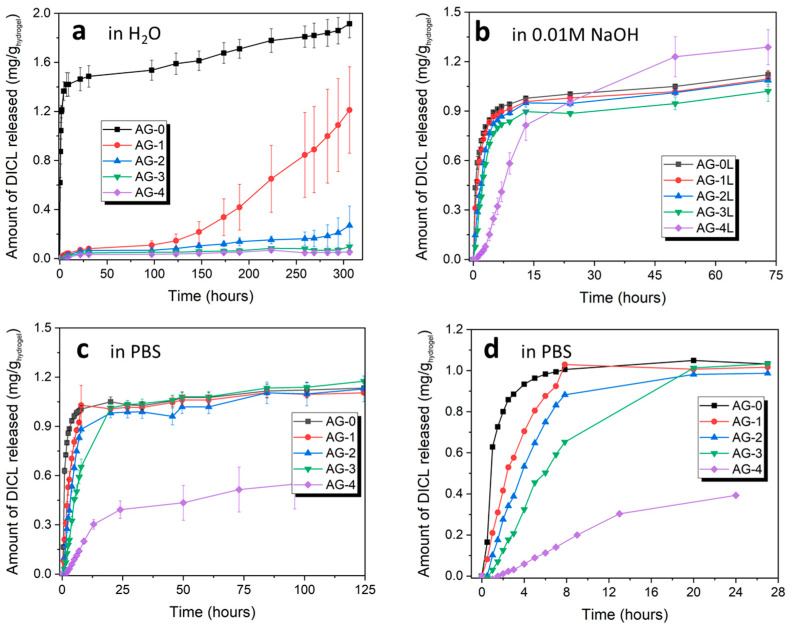
Cumulative release profiles of the studied hydrogels in various media: (**a**) water (pH = 5.5), (**b**) 0.01 M NaOH (pH = 12.0), and (**c**,**d**) PBS (pH = 7.2).

As it can be seen from Figure 5, hydrogels exhibit different release behavior, which depends both on the medium in which diclofenac was released, and on the amount of citric acid added during synthesis. In the case of the release of diclofenac in water, for AG-0 hydrogel, a characteristic burst effect is observed; i.e., the rapid release of a very large amount of the substance in the initial period. The rest of the hydrogels show quite a weak release of diclofenac in this regard; only after about 150 h does DICL start to be released from the AG-1 hydrogel.

The release behavior changes significantly when diclofenac is released with a highly basic solution (0.01 M NaOH). It is released quite quickly from all hydrogels except the AG-4 hydrogel, for which a more sustainable release is observed over the 50 h. For the remaining hydrogels, the largest amount of diclofenac is released during the first 7 h and, thereafter, no increase in the amount of released drug is observed.

When release is tested in PBS buffer, the diclofenac sodium release profiles from individual hydrogels are different, which is clearly seen in Figure 5d, showing the initial 24 h of DICL release. There is a clear relationship between the controlled release of diclofenac and the amount of citric acid added during synthesis and, thus, an increased degree of the cross-linking of the hydrogel. In the case of the pure agarose AG-0 hydrogel, DICL is released very quickly within the first four hours, after which the released amount increases only slightly. In the case of the AG-1 and AG-2 hydrogels, controlled release is observed for the first 7 h, during which almost all of the drug is released, and a plateau is observed after that time. In the case of the AG-3 hydrogel, a controlled release is observed for the first 20 h, after which any further drug release is negligible. Finally, in the case of the AG-4 hydrogel, a controlled release is observed for the entire test period of over 120 h, with a more pronounced release occurring for the first 24 h, and a significantly slower release occurring after 24 h. This hydrogel is also characterized by the smallest amount of released DICL, a significant part of which most likely remained in the structure after the releasing tests, due to the strong cross-linking of this hydrogel.

The obtained results clearly show that the addition of citric acid and, consequently, the hydrogel structure that is formed due to its presence, have a very significant impact on the possibility of controlling the release of drugs via agarose hydrogels.

## 4. Conclusions

In this study, citric acid was successfully introduced into agarose hydrogels, to adjust the mechanical and chemical properties of the final hydrogels, including their water-holding ability, flexibility, and cross-linking efficiency. Visual inspection and rheological tests of the hydrogels confirmed the formation of a homogeneous network in all the hydrogels, although this could be adjusted via control over the interactions between agarose and citric acid in the hydrogel network. The obtained hydrogels provided pH-responsive properties for effective and sustainable drug release over therapeutically relevant time periods (up to 24 h, and often even longer), suggesting that the obtained agarose-based hydrogels could have great potential as drug release systems in biomedical applications.

## Figures and Tables

**Table 1:** Volumes and concentration of the added solutions used to prepare the resulting hydrogels.

Sample Name	Volume of 2% Agarose	Volume of Water	Volume of Citric Acid	Conc. of Citric Acid	AG and CA Content in Dry Mas *
AG-0	4 mL	1 mL	0 mL	---	0.08 g/0.0 g
AG-1	4 mL	0 mL	1 mL	1.0%	0.08 g/0.01 g
AG-2	4 mL	0 mL	1 mL	2.5%	0.08 g/0.025 g
AG-3	4 mL	0 mL	1 mL	5.0%	0.08 g/0.05 g
AG-4	4 mL	0 mL	1 mL	10.0%	0.08 g/0.1 g

* AG, agarose; CA, citric acid.

**Table 2:** The values of the viscoelastic moduli (G′ and G″) and yielding points (γ_L_) for the hydrogels studied.

Sample Name	Storage Modulus, G′ (Pa)	Loss Modulus, G″ (Pa)	Yielding Point, γ_L_ (%)
AG-0	4815 ± 14	338 ± 8	~9
AG-1	6450 ± 18	373 ± 17	~10
AG-2	6463 ± 11	333 ± 5	~10
AG-3	5682 ± 20	355 ± 26	~14
AG-4	5313 ± 30	349 ± 16	~20

## Data Availability

The data presented in this study are available on request from the corresponding author.
